# A national and sub-national metaregression of the trend of insufficient physical activity among Iranian adults between 2001 and 2016

**DOI:** 10.1038/s41598-021-00252-3

**Published:** 2021-11-02

**Authors:** Aida Kamalian, Fatemeh Khosravi Shadmani, Moein Yoosefi, Bahram Mohajer, Farnam Mohebi, Shohreh Naderimagham, Negar Rezaei, Erfan Ghasemi, Mahtab Rouhifard Khalilabad, Bahar Hassanmirzaei, Maryam Selk Ghaffari, Afifeh Khosravi, Ramin Kordi, Farshad Farzadfar

**Affiliations:** 1 Non-Communicable Diseases Research Center, Endocrinology and Metabolism Population Sciences Institute, Tehran University of Medical Sciences, Tehran, Iran; 2 Department of Medicine, Tehran University of Medical Sciences, Tehran, Iran; 3 Research Center for Environmental Determinants of Health (RCEDH), Health Institute, Kermanshah University of Medical Sciences, Kermanshah, Iran; 4 Department of Biostatistics, Faculty of Paramedical Sciences, Shahid Beheshti University of Medical Sciences, Tehran, Iran; 5 Haas School of Business, University of California, Berkeley, CA USA; 6 Endocrinology and Metabolism Research Center, Endocrinology and Metabolism Clinical Sciences Institute, Tehran University of Medical Sciences, Tehran, Iran; 7 Sports Medicine Research Center, Neuroscience Institute, Tehran University of Medical Sciences, Tehran, Iran; 8 Iran Football Medical Assessment and Rehabilitation Center, IFMARC, Tehran, Iran

**Keywords:** Public health, Epidemiology

## Abstract

Insufficient physical activity (IPA) caused approximately 5% of mortalities in 2017 in Iran, almost double its global average. Despite the relatively considerable burden, a knowledge gap exists regarding the trend of IPA in recent years. We described the trend of IPA prevalence utilizing the data from six rounds of STEPwise approach to risk factor Surveillance (STEPS) in Iran. We estimated the physical activity status of Iranian adults from 2006 to 2016 after adjusting for years of schooling, urbanization percentage, and wealth index. We used the spatiotemporal model to interpolate and extrapolate the IPA prevalence for the years in-between the series and from 2001 to 2006, respectively. We used the data of 177,910 participants from six STEPS surveys and found that the national prevalence of IPA had steadily increased over the course of 16 years and had almost doubled in this time period (23.1% in 2001 to 55.4% in 2016). The increase was persistent across all age and gender strata and in every province. Moreover, IPA was more prevalent among women than their male peers regardless of their age category or province of residence. The prevalence of IPA in Khuzestan (highest prevalence) was almost double compared to that in Lorestan (lowest prevalence) in 2016. The IPA prevalence increased considerably and almost doubled in 16 years among Iranian adults, particularly women. Policies need to target IPA as a high priority contributing to the burden of Non-communicable diseases.

## Introduction

Non-communicable diseases (NCDs) are the leading cause of mortality worldwide, with 40.5 million (71%) attributed deaths in 2016^1^. Insufficient physical activity (IPA), considered a major NCD risk factor, is defined as physical activity below the minimum recommended levels proposed by the World Health Organization (WHO). Physical activity below these thresholds accounted for about 2.3 percent of all-cause mortality worldwide in all ages in 2017^1–3^. In 2016, one in four men and one in three women had IPA globally^4^; this was the result of a constant increase of IPA in the past two decades (28.5% in 2001; 27.5% in 2016)^4^. Given that the burden of NCDs falls disproportionately on low- and middle-income countries (LMIC), tackling major NCD risk factors such as IPA effectively in these countries becomes a clear concern^5^.

According to the Global Burden of Diseases (GBD) report in 2017, IPA was responsible for approximately 5% of all-cause mortalities in Iran^2^, resulting in a mortality rate double that of the global average. However, despite being a major health concern, there have been inconsistencies among studies reporting IPA prevalence; from approximately 30% to almost 70%^6^. In the absence of national-level statistics on the IPA trend, the influence of past policies and their future direction are not clear. Moreover, identifying less physically active subpopulations facilitates further modifications of intervention strategies for a better outreach.

In this study, we aimed to report national and sub-national trends of IPA prevalence during a 16-year period from 2001 to 2016 in Iran using the results of six rounds of the STEPwise approach to risk factor Surveillance (STEPS) surveys regarding IPA prevalence in Iran.

## Methods

### Study population and data collection

The data for this study was obtained from NCD risk factors' surveys in Iran. These surveys were conducted based on the standardized approach devised by the WHO, known as STEPS, to monitor NCD risk factors on a national level^7^. A total of seven STEPS surveys have been conducted in Iran (2005, 2006, 2007, 2008, 2009, 2011, 2016). However, the results of the 2005 STEPS survey were excluded from this study due to the inability to calculate questionnaire-based physical activity scores. Participants consisted of Iranian adults from all 31 provinces of Iran. A systematic cluster random sampling frame was used for proportional-to-size sampling from each province's rural and urban areas. All the participants included in this study were aged 18 years or above. All STEPS participants were provided with detailed information regarding the study's objectives and methods. Informed consent was obtained from all STEPS participants. The WHO STEPwise approach to NCDs risk factor surveillance was used in the surveys and interview-based data on demographic and behavioral risk factors was collected in the first step^7^. The protocol for this study has been approved by the ethics committee of the National Institute for Medical Research Development and according the World Medical Association Declaration of Helsinki guidelines.

Data on physical activity (PA) were collected through face-to-face interviews using the Global Physical Activity Questionnaire Version 2 (GPAQ-2)^8^. The GPAQ-2 is developed by the WHO, and collects information on the frequency (days) and duration (minutes/hours) of moderate and vigorous intensity PA in three PA domains of work, transport, and recreation. The analysis protocol of GPAQ-2 was followed during the process of data collection and analysis^8^. Interviews that contained crucial missing data were eliminated from the study based on the GPAQ-2 cleaning guidelines^8^.

### Definition of variables

As mentioned above, the energy expenditure of the participant was estimated based on the duration, intensity, and frequency of PA in a typical week. The unit for measuring PA energy expenditure, Metabolic Equivalent Tasks (MET), is a relative proportion of one's metabolic rate when physically active, divided by his/her resting metabolic rate. Therefore, the total PA in GPAQ-2 was computed as the sum of all MET/minutes/week from moderate-to vigorous-intensity PA performed in the domains of work, transport and recreation^8^. After calculating of the total PA score, each person was classified into low, moderate, or high PA categories based on GPAQ-2 guidelines.

A person classified as Low PA based on the GPAQ-2 criteria is one who has had no or insufficient activity reported and does not meet high and moderate level conditions. This individual falls into the category of insufficient physical activity (IPA) as defined by WHO (METs less than 600)^8^.

### Statistical analysis

Prevalence of IPA was calculated after applying weights to the samples, based on the population size, age, and gender distribution (survey analysis). Means, proportions, and their 95% uncertainty intervals (UI) were expressed by age, gender, and province. Means and 95% UI of METs were also calculated and reported by age, gender, and provincial strata. A type of generalized linear mixed model (GLMM) was created based on the aggregated provincial data from the STEPS to estimate the IPA in timepoints where data was not available and in-between timepoints. In order to improve the estimations' accuracy, several covariates were added to the model, including years of schooling, urbanization percentage, and wealth index. These covariates were computed from the Household Income and Expenditure Surveys (HIES). Wealth index is an instrument that measures the economic status of households and was calculated through the evaluation of household assets by using principal component analysis. The results were categorized into five quintiles, from the poorest to the richest. The spatiotemporal model was used to interpolate IPA prevalence between STEPS survey timelines and extrapolate it from 2001 to 2006. Therefore, the data of IPA for 2001 was estimated based on the model although the STEPS survey was not conducted in 2001. This model takes both temporal and spatial correlations into account in order to reveal continuous and discrete changes in the provinces in this period and to calculate the influence of neighboring spatiotemporal objects on one another. The trend of IPA prevalence was reported in age, gender, and provincial strata in four timepoints, each 5 years apart, during this 15-year period of time. All statistical analyses were calculated and figures were depicted using R. Software version 3.2.1 (Vienna, Austria)^9^.

## Results

A total of 153,882 participants who were 18 years of age or above were interviewed from 2006 to 2016 were included in this study after data cleaning according to GPAQ-2 analysis guidelines. Table 1 demonstrates the number of included participants and the age, gender, and urbanization characteristics of participants in each survey. As demonstrated, the population characteristics of the surveys (age group composition, gender, and urbanization) are rather similar until 2011. The 2011 and 2016 surveys include more participants from both extremes of age groups, more women, and in 2016 a higher number of rural participants in the survey. It is worth mentioning again that prevalence of IPA was calculated after applying weights to the samples, based on the population size, age, and gender distribution and several covariates were added to the model, including years of schooling, urbanization percentage, and wealth index.

**Table 1:** Age, gender, and urbanization characteristics of participants in each STEPS survey.

	2006	2007	2008	2009	2011	2016
**Age**						
Below 24 (%)	5423 (18.53%)	1 (0%)	5673 (19.51%)	5741 (19.66%)	1 (0.01%)	2803 (9.18%)
25–34 (%)	5719 (19.54%)	5740 (22.65%)	5765 (19.82%)	5727 (19.62%)	2349 (22.47%)	7291 (23.87%)
35–44 (%)	5864 (20.03%)	5965 (23.54%)	5826 (20.04%)	5876 (20.12%)	1537 (14.7%)	6495 (21.27%)
45–54 (%)	6002 (20.50%)	5984 (23.62%)	5875 (20.21%)	5885 (20.16%)	1479 (14.15%)	5573 (18.25%)
55–64 (%)	5874 (20.07%)	5788 (22.84%)	5710 (19.64%)	5865 (20.08%)	2187 (20.92%)	4379 (14.34%)
Over 64 (%)	0 (0%)	114 (0.45%)	0 (0%)	0 (0%)	1140 (10.91%)	4000 (13.1%)
Missing (%)	390 (1.33%)	1746 (6.89%)	227 (0.78%)	108 (0.37%)	1760 (16.84%)	0 (0%)
**Gender**						
Number of Women (%)	14,526 (49.62%)	12,432 (49.06%)	14,329 (49.28%)	14,424 (49.39%)	5881 (56.26%)	15,975 (52.31%)
Number of Men (%)	14,746 (50.38%)	12,899 (50.91%)	14,746 (50.72%)	14,756 (50.53%)	4325 (41.38%)	14,566 (47.69%)
Missing (%)	0 (0%)	7 (0.03%)	1 (0%)	22 (0.08%)	247 (2.36%)	0 (0%)
**Urbanization**						
Rural (%)	11,077 (37.84%)	10,360 (40.89%)	11,017 (37.89%)	13,305 (45.56%)	3189 (30.51%)	21,493 (70.37%)
Urban (%)	18,195 (62.16%)	14,974 (59.1%)	18,058 (62.11%)	15,896 (54.43%)	7215 (69.02%)	9048 (29.63%)
Missing (%)	0 (0%)	4 (0.02%)	1 (0%)	1 (0%)	49 (0.47%)	0 (0%)
Total	29,272 (100%)	25,338 (100%)	29,076 (100%)	29,202 (100%)	10,453 (100%)	30,541 (100%)

The STEPS survey 2005 was excluded from the study due to incoherence of its questionnaire with the next surveys. STEPS: STEPwise approach to Surveillance.

**Figure 1:**
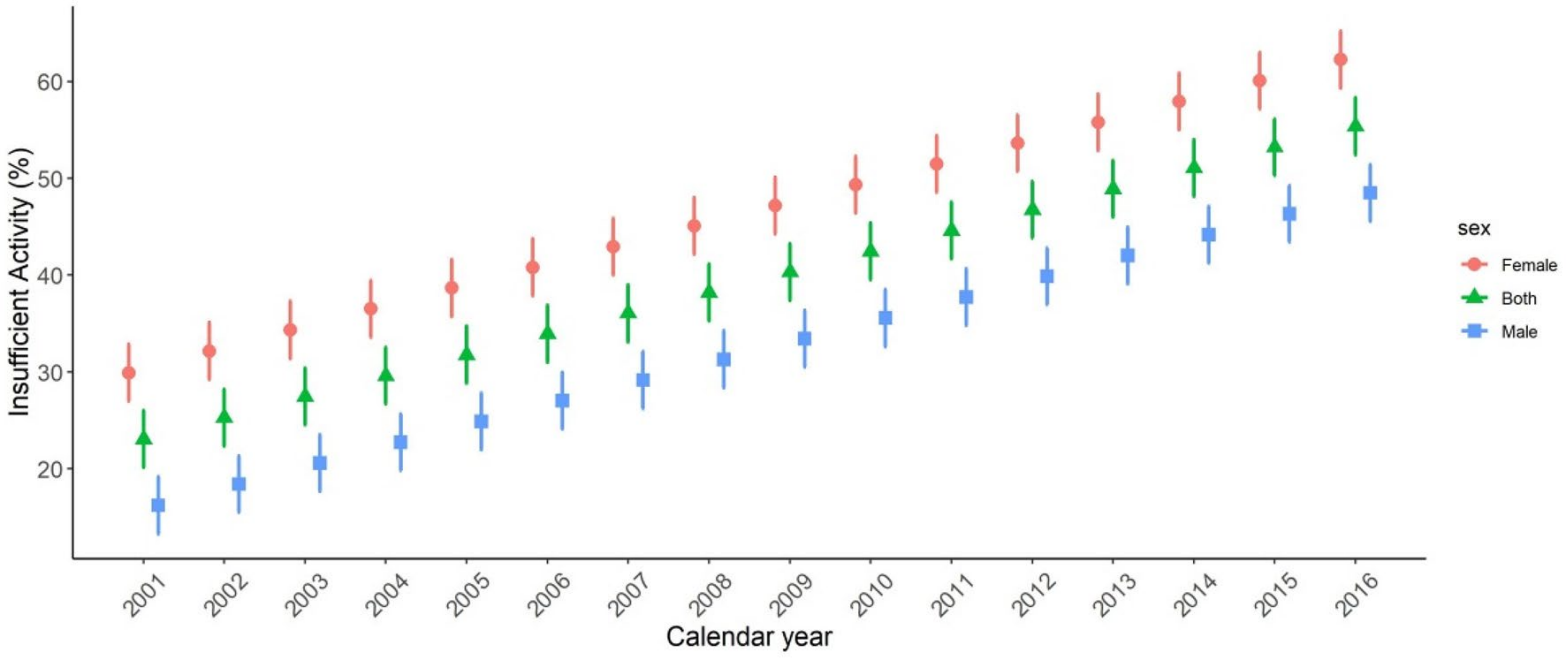
The trend of IPA from 2001 to 2016 is demonstrated below for male, female, and both genders. The lines represent 95% UI of IPA prevalence in each time point. IPA: insufficient physical activity; 95% UI: 95% ucertainty interval.

The prevalence of IPA, defined as the prevalence of low levels of total PA score, steadily increased and approximately doubled in the course of 16 years, rising from the estimated prevalence of 23.1% (95% UI: 20.1–26.0) in 2001 to 33.9% (95% UI: 31.0–36.8) in 2006, 44.6% (95% UI: 41.7–47.5) in 2011, and finally 55.4% (95% UI: 52.4–58.3) in 2016 (Table 2). Figure 1 presents the trend of IPA prevalence in both genders and separately for each gender during the period of 16 years. As Fig. 1 and Tables 2 and 3 demonstrate, the IPA prevalence in 2001, 2006, 2011, and 2016 increases beyond the upper limit of UI compared to previous timepoint in total and in every subcategory (e.g. age, gender, and provincial subgroups).

**Table 2:** The mean IPA prevalence in various age categories.

Age category	Insufficient Physical Activity_2001 (%)			Insufficient Physical Activity_2006 (%)			Insufficient Physical Activity_2011 (%)			Insufficient Physical Activity_2016 (%)		
	Both genders	Female	Male	Both genders	Female	Male	Both genders	Female	Male	Both genders	Female	Male
18–24	17.85 (14.91–20.76)	24.71 (21.76–27.61)	10.99 (8.06–13.92)	28.68 (25.73–31.59)	35.55 (32.6–38.49)	21.81 (18.86–24.7)	39.35 (36.47–42.24)	46.24 (43.33–49.11)	32.47 (29.61–35.38)	50.15 (47.23–53.06)	57.05 (54.16–59.99)	43.25 (40.29–46.14)
25–29	19.99 (17.05–22.93)	26.87 (23.9–29.8)	13.11 (10.21–16.06)	30.83 (27.94–33.77)	37.7 (34.8–40.64)	23.96 (21.08–26.9)	41.51 (38.59–44.42)	48.39 (45.46–51.32)	34.63 (31.73–37.51)	52.29 (49.34–55.23)	59.17 (56.21–62.13)	45.41 (42.47–48.32)
30–34	20.91 (18–23.83)	27.75 (24.82–30.71)	14.07 (11.17–16.96)	31.74 (28.82–34.65)	38.62 (35.7–41.53)	24.85 (21.95–27.77)	42.43 (39.49–45.33)	49.3 (46.38–52.21)	35.56 (32.61–38.45)	53.2 (50.29–56.13)	60.08 (57.16–63)	46.32 (43.42–49.26)
35–39	21.68 (18.77–24.64)	28.54 (25.63–31.49)	14.82 (11.91–17.79)	32.52 (29.62–35.45)	39.39 (36.47–42.34)	25.65 (22.77–28.55)	43.2 (40.28–46.13)	50.08 (47.15–53.02)	36.31 (33.41–39.23)	53.98 (51.06–56.9)	60.89 (57.95–63.79)	47.07 (44.17–50)
40–44	22.46 (19.53–25.38)	29.33 (26.38–32.27)	15.59 (12.67–18.5)	33.3 (30.39–36.2)	40.19 (37.29–43.12)	26.42 (23.49–29.29)	43.97 (41.05–46.88)	50.85 (47.93–53.76)	37.09 (34.17–40)	54.76 (51.84–57.71)	61.64 (58.7–64.6)	47.87 (44.97–50.82)
45–49	23.31 (20.37–26.25)	30.18 (27.26–33.11)	16.44 (13.48–19.38)	34.13 (31.22–37.06)	40.99 (38.09–43.94)	27.27 (24.35–30.19)	44.83 (41.91–47.75)	51.72 (48.78–54.64)	37.95 (35.04–40.85)	55.62 (52.67–58.52)	62.5 (59.55–65.42)	48.74 (45.78–51.63)
50–54	24.26 (21.37–27.22)	31.12 (28.24–34.06)	17.41 (14.5–20.39)	35.11 (32.2–38.03)	41.98 (39.08–44.9)	28.23 (25.33–31.16)	45.77 (42.86–48.71)	52.66 (49.76–55.6)	38.89 (35.97–41.82)	56.56 (53.66–59.47)	63.45 (60.58–66.37)	49.66 (46.75–52.57)
55–59	25.4 (22.49–28.32)	32.25 (29.35–35.19)	18.55 (15.63–21.44)	36.22 (33.3–39.17)	43.11 (40.17–46.06)	29.34 (26.43–32.28)	46.92 (44–49.83)	53.8 (50.86–56.71)	40.03 (37.14–42.94)	57.7 (54.76–60.6)	64.59 (61.64–67.51)	50.81 (47.89–53.7)
60–64	26.69 (23.76–29.61)	33.57 (30.59–36.47)	19.81 (16.93–22.76)	37.54 (34.64–40.44)	44.41 (41.53–47.31)	30.68 (27.74–33.56)	48.21 (45.31–51.13)	55.09 (52.17–57.99)	41.34 (38.45–44.27)	58.99 (56.08–61.92)	65.88 (62.95–68.82)	52.11 (49.2–55.03)
65–70	28.21 (25.3–31.17)	35.07 (32.14–38.03)	21.35 (18.46–24.31)	39.06 (36.15–41.98)	45.93 (43.01–48.87)	32.2 (29.29–35.1)	49.73 (46.82–52.64)	56.61 (53.67–59.51)	42.85 (39.97–45.77)	60.52 (57.58–63.47)	67.41 (64.44–70.38)	53.63 (50.71–56.55)
18–70	23.08 (20.15–26.01)	29.94 (27.01–32.87)	16.21 (13.3–19.15)	33.91 (31–36.83)	40.79 (37.87–43.72)	27.04 (24.13–29.95)	44.59 (41.68–47.51)	51.47 (48.55–54.39)	37.71 (34.81–40.62)	55.38 (52.45–58.3)	62.27 (59.33–65.2)	48.49 (45.57–51.4)

*IPA* insufficient physical activity, *95% UI* 95% uncertainty interval.

**Table 3:** The mean IPA prevalence in the provincial strata.

Province	Insufficient Physical Activity_2001 (%)			Insufficient Physical Activity_2006 (%)			Insufficient Physical Activity_2011 (%)			Insufficient Physical Activity_2016 (%)		
	Both genders	Female	Male	Both genders	Female	Male	Both genders	Female	Male	Both genders	Female	Male
Alborz	20.29 (17.1–23.47)	27.22 (24.04–30.38)	13.36 (10.16–16.55)	31.09 (27.9–34.14)	38 (34.78–41.13)	24.18 (21.02–27.16)	41.74 (38.67–44.83)	48.61 (45.5–51.75)	34.87 (31.85–37.92)	52.53 (49.44–55.6)	59.39 (56.28–62.46)	45.68 (42.61–48.74)
Ardabil	26.07 (23.25–28.97)	32.9 (30.08–35.78)	19.24 (16.41–22.16)	36.98 (34.19–39.79)	43.86 (40.98–46.68)	30.1 (27.41–32.91)	47.7 (44.85–50.49)	54.57 (51.72–57.35)	40.83 (37.99–43.62)	58.5 (55.66–61.36)	65.39 (62.56–68.23)	51.62 (48.76–54.49)
Azerbaijan, East	20.42 (17.58–23.26)	27.28 (24.47–30.14)	13.56 (10.69–16.38)	31.25 (28.44–34.08)	38.13 (35.33–41)	24.36 (21.56–27.16)	41.91 (39.04–44.76)	48.8 (45.93–51.66)	35.03 (32.15–37.86)	52.7 (49.85–55.52)	59.57 (56.76–62.43)	45.83 (42.94–48.61)
Azerbaijan, West	20.4 (17.53–23.23)	27.26 (24.42–30.09)	13.54 (10.64–16.37)	31.27 (28.42–34.08)	38.16 (35.31–40.96)	24.39 (21.53–27.19)	41.95 (39.16–44.76)	48.85 (46.03–51.7)	35.05 (32.29–37.81)	52.76 (49.91–55.65)	59.68 (56.78–62.6)	45.84 (43.05–48.69)
Bushehr	30.68 (27.87–33.52)	37.57 (34.77–40.37)	23.79 (20.96–26.67)	41.5 (38.64–44.32)	48.4 (45.55–51.3)	34.59 (31.74–37.34)	52.19 (49.38–55.03)	59.08 (56.21–61.9)	45.3 (42.56–48.16)	63.02 (60.14–65.91)	69.79 (66.9–72.67)	56.25 (53.37–59.14)
Chahar Mahaal and Bakhtiari	19.18 (16.35–21.98)	26.08 (23.24–28.78)	12.28 (9.47–15.18)	30 (27.15–32.81)	36.89 (34.06–39.68)	23.11 (20.24–25.94)	40.65 (37.84–43.46)	47.53 (44.72–50.34)	33.77 (30.95–36.58)	51.34 (48.48–54.18)	58.17 (55.31–61.01)	44.5 (41.65–47.35)
Fars	33.56 (30.75–36.39)	40.42 (37.64–43.24)	26.69 (23.85–29.55)	44.37 (41.56–47.17)	51.21 (48.43–54.05)	37.53 (34.7–40.29)	55.05 (52.21–57.88)	61.91 (59.1–64.75)	48.18 (45.31–51.01)	65.79 (63–68.64)	72.69 (69.9–75.54)	58.9 (56.11–61.73)
Gilan	18.93 (15.96–21.88)	25.8 (22.84–28.76)	12.07 (9.08–15.01)	29.78 (26.86–32.66)	36.66 (33.73–39.53)	22.9 (19.99–25.79)	40.55 (37.65–43.47)	47.44 (44.54–50.4)	33.66 (30.77–36.54)	51.48 (48.63–54.34)	58.33 (55.45–61.18)	44.63 (41.82–47.49)
Golestan	27.55 (24.63–30.47)	34.4 (31.46–37.37)	20.7 (17.81–23.57)	38.33 (35.4–41.28)	45.2 (42.35–48.19)	31.46 (28.44–34.38)	49 (46.04–51.94)	55.89 (52.92–58.84)	42.1 (39.15–45.04)	59.72 (56.71–62.64)	66.68 (63.67–69.6)	52.77 (49.74–55.68)
Hamadan	18.58 (15.76–21.49)	25.44 (22.57–28.39)	11.71 (8.95–14.58)	29.43 (26.59–32.27)	36.3 (33.47–39.14)	22.56 (19.71–25.39)	40.09 (37.23–42.93)	46.98 (44.18–49.79)	33.2 (30.27–36.07)	50.87 (48.01–53.7)	57.78 (54.93–60.64)	43.96 (41.09–46.76)
Hormozgan	33.4 (30.45–36.42)	40.23 (37.23–43.29)	26.57 (23.67–29.55)	44.21 (41.2–47.23)	51.04 (48–54.12)	37.38 (34.4–40.33)	54.91 (51.92–57.89)	61.74 (58.76–64.72)	48.08 (45.08–51.06)	65.72 (62.72–68.74)	72.57 (69.61–75.63)	58.87 (55.84–61.84)
Ilam	17.24 (14.37–20.08)	24.11 (21.24–26.95)	10.37 (7.51–13.2)	28.06 (25.27–30.93)	34.96 (32.17–37.82)	21.17 (18.36–24.04)	38.74 (35.93–41.59)	45.66 (42.84–48.51)	31.81 (29.03–34.66)	49.53 (46.64–52.43)	56.45 (53.55–59.33)	42.62 (39.74–45.52)
Isfahan	26.77 (23.73–29.81)	33.65 (30.61–36.74)	19.88 (16.85–22.88)	37.6 (34.63–40.66)	44.49 (41.53–47.5)	30.71 (27.72–33.81)	48.31 (45.32–51.34)	55.21 (52.19–58.23)	41.42 (38.45–44.45)	59.16 (56.12–62.17)	66.05 (63–69.06)	52.26 (49.25–55.29)
Kerman	27.61 (24.74–30.45)	34.44 (31.52–37.29)	20.77 (17.96–23.6)	38.37 (35.58–41.2)	45.21 (42.45–48.07)	31.52 (28.72–34.33)	48.94 (46.07–51.85)	55.79 (52.89–58.71)	42.1 (39.25–45)	59.58 (56.61–62.44)	66.45 (63.49–69.3)	52.71 (49.73–55.57)
Kermanshah	15.82 (12.97–18.65)	22.69 (19.8–25.49)	8.95 (6.15–11.82)	26.67 (23.79–29.57)	33.54 (30.66–36.41)	19.81 (16.93–22.72)	37.38 (34.56–40.22)	44.27 (41.41–47.12)	30.49 (27.71–33.32)	48.19 (45.34–51.08)	55.13 (52.32–58)	41.24 (38.36–44.16)
Khorasan, North	19.68 (16.74–22.7)	26.5 (23.57–29.54)	12.86 (9.9–15.85)	30.5 (27.55–33.48)	37.33 (34.37–40.33)	23.67 (20.73–26.63)	41.16 (38.19–44.13)	48.02 (45–51.04)	34.29 (31.38–37.21)	51.93 (48.95–54.86)	58.83 (55.87–61.79)	45.03 (42.02–47.93)
Khorasan, Razavi	19.52 (16.7–22.36)	26.36 (23.57–29.2)	12.68 (9.84–15.52)	30.42 (27.54–33.27)	37.29 (34.47–40.13)	23.55 (20.61–26.41)	41.08 (38.26–43.9)	47.95 (45.11–50.77)	34.21 (31.4–37.03)	51.86 (49.03–54.69)	58.78 (55.9–61.58)	44.93 (42.16–47.79)
Khorasan, South	16.27 (13.31–19.26)	23.09 (20.12–26.07)	9.46 (6.5–12.45)	27.05 (24.08–30.05)	33.89 (30.96–36.91)	20.22 (17.2–23.19)	37.69 (34.75–40.66)	44.56 (41.57–47.54)	30.83 (27.92–33.78)	48.49 (45.45–51.44)	55.38 (52.3–58.37)	41.59 (38.59–44.5)
Khuzestan	46.37 (43.54–49.24)	53.26 (50.37–56.1)	39.49 (36.72–42.37)	57.21 (54.38–60.1)	64.09 (61.25–66.95)	50.33 (47.51–53.25)	67.88 (65.01–70.69)	74.76 (71.88–77.58)	61.01 (58.14–63.8)	78.66 (75.75–81.54)	85.57 (82.66–88.46)	71.75 (68.84–74.61)
Kohgiluyeh and Boyer-Ahmad	9.87 (6.8–12.93)	16.72 (13.64–19.81)	3.02 (0.00–6.06)	20.74 (17.77–23.78)	27.6 (24.63–30.66)	13.87 (10.91–16.9)	31.55 (28.56–34.47)	38.43 (35.43–41.38)	24.66 (21.69–27.55)	42.4 (39.46–45.34)	49.36 (46.39–52.32)	35.45 (32.52–38.36)
Kurdistan	15.62 (12.79–18.5)	22.47 (19.65–25.34)	8.78 (5.93–11.66)	26.54 (23.67–29.35)	33.41 (30.53–36.22)	19.68 (16.81–22.48)	37.28 (34.45–40.09)	44.19 (41.34–47.04)	30.36 (27.56–33.15)	48.09 (45.29–50.95)	55.02 (52.22–57.9)	41.16 (38.35–43.99)
Lorestan	7.31 (4.48–10.19)	14.16 (11.34–16.99)	0.45 (0.00–3.39)	18.12 (15.28–20.98)	24.98 (22.11–27.85)	11.26 (8.45–14.1)	28.75 (25.89–31.6)	35.62 (32.74–38.5)	21.87 (19.04–24.71)	39.53 (36.62–42.36)	46.41 (43.52–49.23)	32.65 (29.73–35.49)
Markazi	18.47 (15.67–21.26)	25.33 (22.55–28.14)	11.61 (8.79–14.38)	29.47 (26.64–32.28)	36.32 (33.51–39.12)	22.61 (19.77–25.44)	40.24 (37.45–43.06)	47.09 (44.31–49.89)	33.38 (30.59–36.22)	51.15 (48.3–53.99)	57.98 (55.13–60.82)	44.31 (41.47–47.16)
Mazandaran	14.47 (11.51–17.43)	21.32 (18.37–24.31)	7.62 (4.65–10.55)	25.2 (22.26–28.19)	32.06 (29.13–35.1)	18.33 (15.4–21.28)	35.84 (32.87–38.81)	42.71 (39.74–45.64)	28.98 (25.99–31.97)	46.57 (43.6–49.62)	53.45 (50.49–56.53)	39.7 (36.7–42.71)
Qazvin	26 (23.17–28.83)	32.84 (30.05–35.68)	19.15 (16.28–21.97)	36.9 (34.02–39.77)	43.8 (40.94–46.62)	30.01 (27.1–32.92)	47.66 (44.82–50.53)	54.52 (51.67–57.37)	40.8 (37.96–43.69)	58.53 (55.73–61.38)	65.37 (62.58–68.2)	51.7 (48.87–54.56)
Qom	33.42 (30.15–36.69)	40.36 (36.99–43.66)	26.49 (23.3–29.72)	44.27 (41.02–47.52)	51.2 (47.89–54.52)	37.34 (34.14–40.52)	54.9 (51.69–58.15)	61.84 (58.6–65.06)	47.96 (44.77–51.23)	65.61 (62.4–68.78)	72.54 (69.3–75.72)	58.67 (55.5–61.84)
Semnan	27.66 (24.74–30.61)	34.5 (31.62–37.45)	20.83 (17.85–23.76)	38.5 (35.63–41.39)	45.37 (42.47–48.22)	31.63 (28.78–34.56)	49.14 (46.28–52)	55.99 (53.15–58.83)	42.29 (39.41–45.17)	59.88 (57.03–62.75)	66.73 (63.87–69.6)	53.03 (50.18–55.89)
Sistan and Baluchistan	33.38 (30.43–36.36)	40.18 (37.2–43.15)	26.58 (23.65–29.56)	44.21 (41.26–47.14)	51.05 (48.06–54.01)	37.37 (34.45–40.27)	54.85 (51.84–57.85)	61.72 (58.72–64.69)	47.98 (44.97–51)	65.62 (62.62–68.65)	72.54 (69.52–75.6)	58.7 (55.73–61.71)
Tehran	23.32 (19.97–26.65)	30.24 (26.8–33.57)	16.41 (13.14–19.74)	34.06 (30.84–37.34)	40.96 (37.68–44.34)	27.17 (24–30.34)	44.73 (41.52–47.91)	51.6 (48.39–54.8)	37.85 (34.65–41.01)	55.5 (52.36–58.7)	62.38 (59.2–65.62)	48.61 (45.53–51.77)
Yazd	32.82 (29.89–35.81)	39.71 (36.78–42.66)	25.94 (22.99–28.96)	43.63 (40.7–46.59)	50.51 (47.58–53.46)	36.75 (33.81–39.72)	54.26 (51.26–57.18)	61.18 (58.16–64.07)	47.34 (44.36–50.3)	64.95 (62.02–67.92)	71.89 (68.96–74.88)	58.01 (55.08–60.97)
Zanjan	14.68 (11.87–17.52)	21.55 (18.72–24.41)	7.81 (5.02–10.63)	25.59 (22.76–28.44)	32.46 (29.66–35.27)	18.72 (15.86–21.61)	36.26 (33.34–39.21)	43.14 (40.24–46.06)	29.38 (26.45–32.36)	47.02 (44.06–49.99)	53.9 (50.95–56.92)	40.14 (37.18–43.07)
National	23.08 (20.15–26.01)	29.94 (27.01–32.87)	16.21 (13.3–19.15)	33.91 (31–36.83)	40.79 (37.87–43.72)	27.04 (24.13–29.95)	44.59 (41.68–47.51)	51.47 (48.55–54.39)	37.71 (34.81–40.62)	55.38 (52.45–58.3)	62.27 (59.33–65.2)	48.49 (45.57–51.4)

*IPA* insufficient physical activity, *95% UI* 95% uncertainty interval.

Although the increase of IPA prevalence was ubiquitous in both genders, it was persistently more prevalent in women. Insufficient PA prevalence was estimated to be 29.9% (95% UI: 27.0–32.9) in women vs. 16.2% (95% UI: 13.3–19.1) in men in 2001 and 62.3% (95% UI: 59.3–65.2) in women vs. 48.5% (95% UI: 45.6–51.4) in men in 2016 (The data for the years in between are shown in Fig. 1 and Table 2).

**Figure 2:**
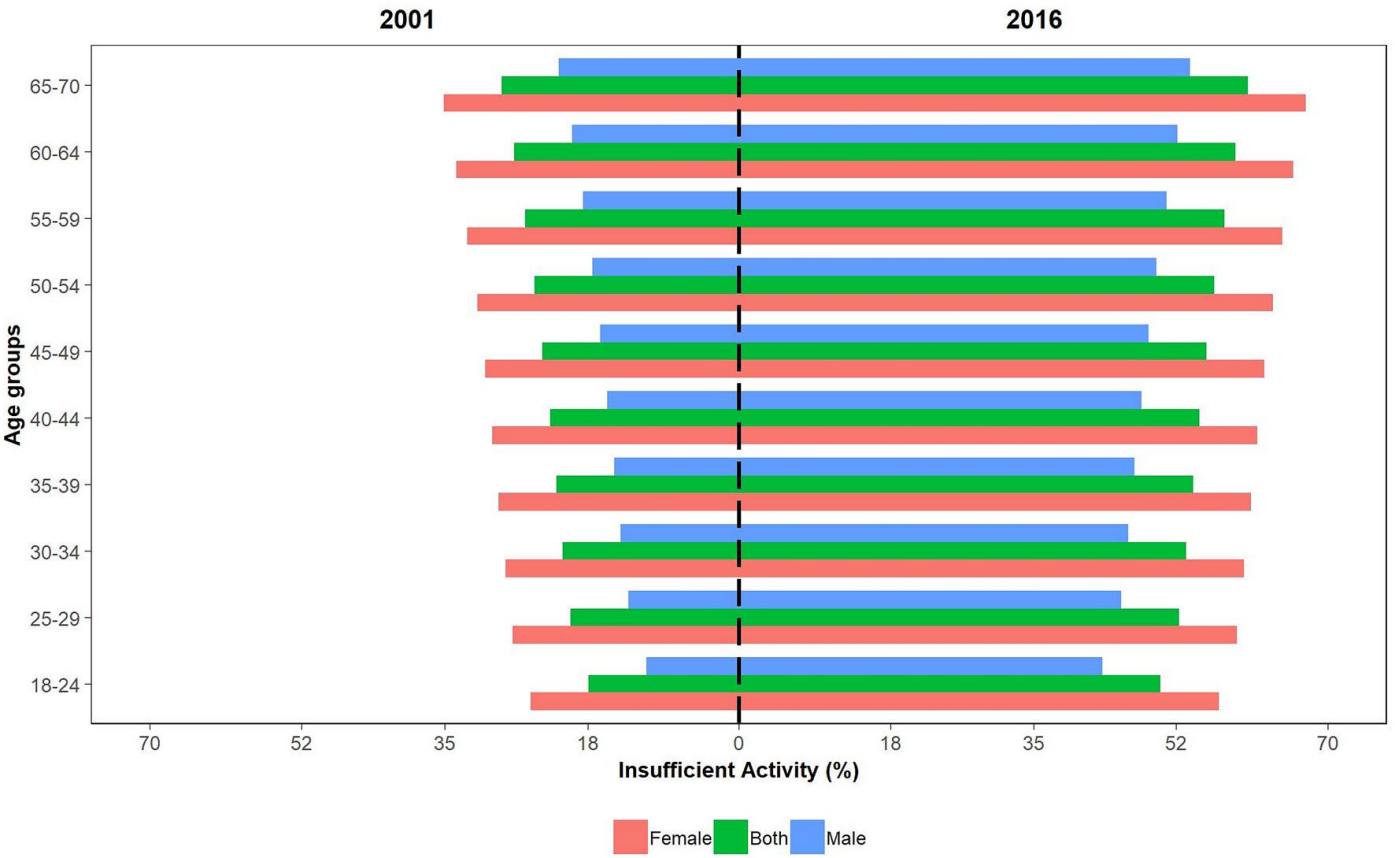
The prevalence of IPA in 2001 and 2016 is demonstrated below for male, female, and both genders. IPA: insufficient physical activity; 95% UI: 95% uncertainty interval.

While IPA prevalence increased in all age group strata of Iranian adults, the lowest percentage of inactive adults belonged to the 18–24 years age group (17.8% (95% UI: 14.9–20.8) in 2001; 50.1% (95% UI: 47.2–53.1) in 2016). Insufficient PA was most prevalent among the eldest age group (65–70) with 28.2% (95% UI: 25.3–31.2) inactive adults in 2001 and 60.5% (95% UI: 57.6–63.5) in 2016. A more detailed report of the results in age group strata is shown in Table 2 and Fig. 2. Moreover, women had a higher percentage of inactive adults in all age group strata in every time point reported (Table 2 and Fig. 2), while the age group strata with the highest and lowest prevalence of IPA remained the same in both genders (18–24 years the lowest and 65–70 years the highest).

**Figure 3:**
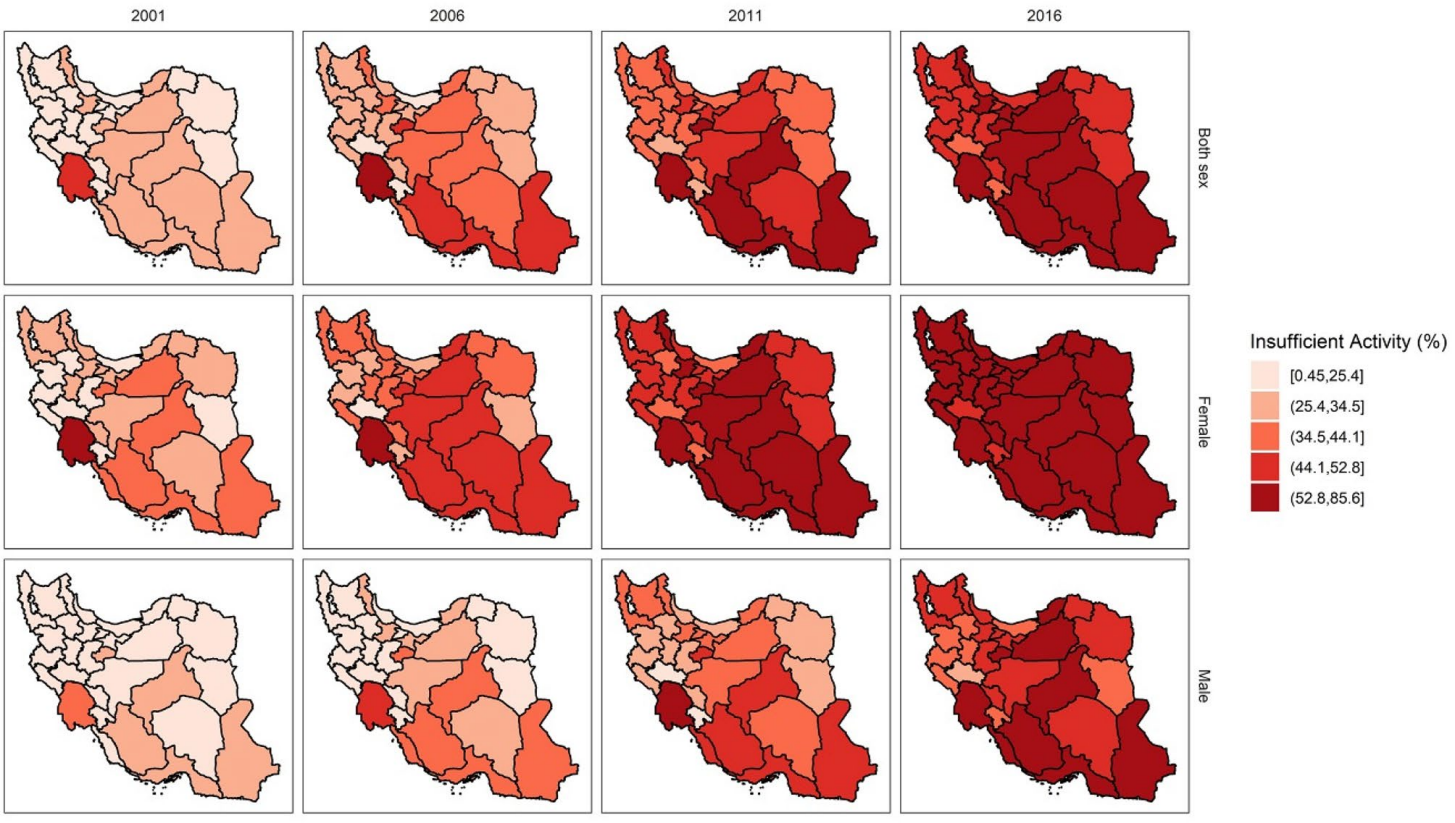
The subnational geographical distributon of IPA prevalence is demonstrated in four time points (2001, 2006, 2011, 2016) for male, female, and both genders. IPA: insufficient physical activity.

Among the 31 provinces studied, Khuzestan consistently had the highest prevalence of inactive adults over the years (both genders: 46.4% (95% UI: 43.5–49.2) in 2001 and 78.7% (95% UI: 75.7–81.5) in 2016; women: 53.3% (95% UI: 50.4–56.1) in 2001 and 85.6% (95% UI: 82.7–88.5) in 2016; men: 39.5% (95% UI: 36.7–42.4) in 2001 and 71.7% (95% UI: 68.8–74.6) in 2016). Conversely, Lorestan was found to be the province with the lowest percentage of IPA among its residents (both genders: 7.3% (95% UI: 4.5–10.2) in 2001 and 39.5% (95% UI: 36.6–42.4) in 2016; women: 14.2% (95% UI: 11.3–17.0) in 2001 and 46.4% (95% UI: 43.5–49.2) in 2016; men: 0.4% (95% UI: 0.0–3.4) in 2001 and 32.6% (95% UI: 29.7–35.5) in 2016). A detailed report on the provincial stratification of IPA prevalence in both genders is provided in Table 3. As shown in Fig. 3 and Table 3, the IPA prevalence increased in all of the provincial strata during the 16-year period. Moreover, women had a higher percentage of inactive adults in every province across all time points.

## Discussion

This study showed that the prevalence of insufficiently physically active Iranian adults above 18 had approximately doubled from 2001 to 2016, and that more than half the population (about 55%) had IPA in 2016. The increase was observed regardless of the population stratifications based on gender, age group, or the province of residence, with the lowest IPA prevalence observed in the youngest age group (18 to 24 years). In every time point studied, women had a higher percentage of adults with IPA, regardless of their age group or province of residence. The investigation of the subnational geographical distribution of IPA prevalence displayed the same increasing trend across the country despite varying levels of IPA in different provinces, and singled out Khuzestan as the province with the highest and Lorestan with the lowest percentage of IPA in 2016. An approximate thirty-percent difference was sustained along these years, although the IPA prevalence in Khuzestan was six times higher in 2001, it decreased to about two times higher percentage in 2016. Considering that the IPA rates at each time point exceeds the upper limit of UI in the previous time point, this increase is statistically significant.

The latest study in 2011 shows that Iran, as a LMIC, has a 21.5% prevalence of IPA which was significantly higher than the previous national surveillance results in 2007 (about 15%)^10^. However, Esteghamati et al. reported the national IPA statistics in 2011 to be 40% in the low physical activity category and 15% with no physical activity at all^11^. Moreover at the same year, Sahebkar et al. reported that 44.8% of Iranian adults are categorized in the low physical activity level based on a Persian version of Global Physical Activity Questionnaire^12^. The national IPA prevalence data reported by this paper were very similar to studies that had utilized the same measurement tools and studied the same age categories at a similar time period^10,13^. Not surprisingly, our results were almost identical to those in which STEPs survey data were used to calculate IPA prevalence^10,12,14^. A unanimous consensus was observed on the higher IPA prevalence of women in Iran regardless of the measurement tools, studied time point, and sample size^15^. Other Middle Eastern countries demonstrated similar or even higher numbers of IPA prevalence, which might point to a larger geographical/cultural underlying risk factor for IPA^16^. For instance, a literature review on the IPA status of adults in Saudi Arabia has shown IPA to range from 26 to 85% among men and from 43 to 91% among women^17^. The total IPA prevalence was reported to be 67.6% according to the STEPS 2005 conducted in Saudi Arabia^18^. This number was 45.9% in Qatar based on STEPS 2012, 56.0% for Turkey based on the World Health Survey (2002–2003), and 30.1% for Oman according to STEPS 2006^18^. These numbers indicate a more or less similar IPA situation in the region with an unequal involvement of women. However, the recent IPA trend in Iran has not been observed globally. Guthold et al. reported a plateau in the trend of IPA globally from 2001 to 2015^4^, which is far from the observed increase in the trend of IPA in Iran and further accentuates the need to address this health issue in Iran and the region.

This trend can be mostly explained by the societal changes in the observed period. The World Bank reports that the urbanized population of Iran has grown from 64.7% in 2001 to 73.9% in 2016^19^, which naturally results in the replacement of physically demanding jobs (e.g. farming) with less demanding ones such as office work^20^. Moreover, the shift of transportation methods to more mechanized vehicles has reduced the commuting physical activity as well^21^. Regarding the reported gender gap, the relationship between gender and physical activity is widely recognized^4^. The WHO's Global Health Observatory reports that the prevalence of IPA in women was on average 9% more than their male peers in 2016^22^. According to this paper, the gender gap is even wider in Iran (almost as high as 13%) which only highlights the importance of addressing the gender issue. Studies conducted in socio-cultural settings similar to Iran have reported several inhibiting factors underlying the mentioned pattern: the limited number of facilities available exclusively to women and a lack of encouragement, peer support, and role-models were among them^16^. Our data also showed that one is more likely to be inactive as one ages, which is a rather consistent finding in the literature^23^. As one ages, factors such as career preoccupation and parenthood may affect the level of PA^24^. Moreover, the increased IPA prevalence in the elderly might be due to psychosocial attributes (e.g. lack of motivation) or health problems of this age category (e.g. lower extremity joint pain)^25^. Apart from age and gender, geography seems to affect sedentary behavior. Previous studies have noted various geographical determinants including the urbanization of the region to play a key role in the observed disparity^26^. Interestingly, a similarity was witnessed between the map of IPA in Iran and the map of provinces' urbanization levels in 2011^27^. This similarity is in line with previous literature and suggests urbanization as a determinant of IPA levels^20^. However, further studies are needed to investigate and determine the significance of this similarity.

For the first time, this paper has reported the results of six series of WHO STEPS surveys for physical inactivity prevalence in Iran from 2001 to 2016 in various age group, gender, and provincial strata. Due to the considerable sample size of this study, investigation of IPA prevalence in a 16-year period, and cross-national geographical distribution of the sample populations, the results of this paper provide reliable information about the recent status of IPA in Iran and its trend in the past years.

The limitations that need to be taken into account regarding this paper include the results of the self-reported PA questionnaires that were prone to recall bias. Both underestimation and overestimation of PA are common in self-reported questionnaires. However, GPAQ-2 is found to be relatively reliable among its counterparts despite its inefficiencies in measuring metabolic and vascular disease risk factors^28^. Moreover, this study would have benefited from a more specific categorization of PA in work, transport, and recreation domains, allowing a more detailed look into the alterations of various categories of physical inactivity during this period. Furthermore, the IPA reliable data was not available before STEPS 2006 and in-between each survey. The data for the time points in-between each survey was obtained by interpolations and extrapolations provided by earlier data from 2001 to 2006.

Considering the notably increasing trend of IPA in Iran, the next appropriate steps seem to be identifying obstacles the less active subpopulations face and modifying the intervention strategies accordingly. The scope and nature of the strategies that must be utilized are stated in WHO's "review of best practice in interventions to promote physical activity in developing countries"^29^. These interventions must incorporate several different intervention strategies including raising awareness about the health benefits of PA, organizing local exercise programs, creating facilities for PA, and designing the interventions in an all-inclusive manner in various domains (e.g. schools, the workplace, health centers, and community). In order for the interventions to engage people from all settings, it is necessary to be aware of the existent obstacles and disparities in various subpopulations and plan the strategies accordingly. Needless to say, building a social structure and neighborhood environment that encourages PA for women is of paramount importance according to the presented data. Health promotion interventions for women can include creating culturally appropriate PA facilities, developing safe neighborhood environments appropriate for more community-based approaches such as walking groups, and raising awareness and social support through campaigns^16^. An example of the successful policy implementation to tackle the issue of IPA is the Turkish Healthy Nutrition and Active Life Programme (HNAP) implemented from 2010 to 2014 which targets obesity through dietary factors and physical activity. The HNAP planned to improve PA by improving the “cyclability” and “walkability” of the built environment, physical education in schools, and PA in the workplace^30^. The WHO has announced the success of this program in increasing population knowledge and adoption of healthy lifestyle habits^31^. Considering the economic and geographical status similarity of Turkey and Iran, adopting the same policies with a focus on Iran's specific challenges (e.g. gender gap) might be beneficial.

Further studies are necessary to follow the IPA trend in the coming years in Iran and investigate the existence of its correlation with factors such as urbanization. Moreover, a more detailed investigation of the IPA trend in the three domains of work, transport, and recreation could be considered a noteworthy endeavor.

## Conclusion

The trend of insufficient physical activity continued to rise during the 16-year observation period in Iran across all age groups, genders, and provinces and the prevalence eventually doubled. This study further illuminates the existent disparity between men and women, with IPA being considerably more prevalent in women and the central provinces. Policies focusing on the psychosocial and environmental barriers of subpopulations with higher IPA prevalence must be on the agendas of the policymakers. Further studies in future are required to follow up this trend in the coming years.

### Clarification

I certify that this manuscript is original, has not yet been published and is not under consideration for publication elsewhere. All authors have made a significant contribution to the manuscript and accept the responsibility for the study protocol and the presented results. The protocol for this study has been approved by the Ethical Committee of the National Institute for Medical Research Development (NIMAD), Tehran, Iran (ID:IR.NIMAD.REC.1394.032) and according the World Medical Association Declaration of Helsinki guidelines. No conflict of interest exists in relation to the submitted manuscript.
